# From wilderness to cohabitation: Literature review on human presence in environmental ethics

**DOI:** 10.1007/s13280-026-02377-z

**Published:** 2026-04-29

**Authors:** Teea Kortetmäki, Gonzalo Cortés Capano

**Affiliations:** 1 Department of Social Sciences and Philosophy, University of Jyväskylä, PO BOX 35, 40014 Jyväskylä, Finland; 2 School of Resource Wisdom, University of Jyväskylä, PO BOX 35, 40014 Jyväskylä, Finland; 3 Social-Ecological Interactions in Agricultural Systems, University of Kassel and University of Göttingen, Kassel and Göttingen, Germany

**Keywords:** Coexistence, Genealogical analysis, Human-nature relations, Land stewardship, Relational ethics, Values

## Abstract

Land use and biodiversity conservation are increasingly recognized as ethical and value-laden topics rather than merely ecological or socio-technical problems. While this has increased multidisciplinary and participatory approaches in research, explicit engagement with environmental ethics, where those questions are scrutinized though an ethical lens, remains limited. To address this need, we reviewed how the environmental ethics literature defines and characterizes active human presence across different environments, how it frames human impacts on nonhumans, and the assumptions and normative implications articulated by these perspectives. Using a narrative approach, we identify four main narratives from environmental ethics on human and nonhuman coexistence: wilderness, rural stewardship, urban cohabitation, and relational narratives. By synthesizing these narratives, our findings offer conceptual tools that can support sustainability researchers, practitioners, and policymakers reflect more critically on the ethical dimensions of land-use decisions and better navigate contested visions of desirable landscape futures.

****Graphical abstract**:**

**Figure:**
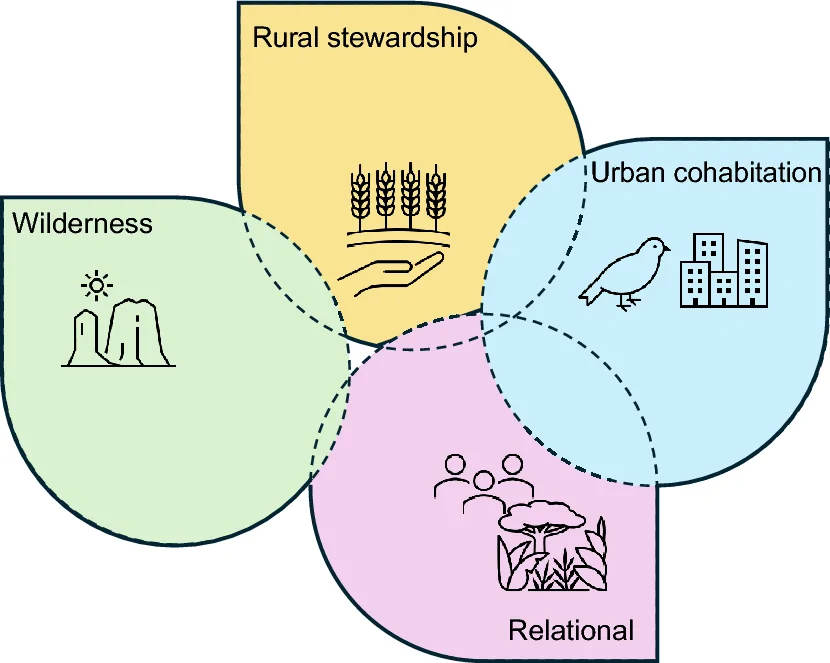

**Supplementary Information:**

The online version contains supplementary material available at https://doi.org/10.1007/s13280-026-02377-z.

## Introduction

Land use and land management practices are critically important for biodiversity conservation and planetary wellbeing because they are among the key drivers of biodiversity loss (IPBES 2018; Semenchuk et al. 2022; Cabernard et al. 2024). This has evoked foundational questions regarding sustainable land use strategies that are often captured as a paradigmatic debate between ‘land sharing’ and ‘land sparing’ approaches (Law and Wilson 2015; Grass et al. 2019). While scholarship increasingly recognizes that both sharing and sparing are context-dependent and often complementary strategies involving complex socio-political dimensions (Fischer et al. 2014; Crespin and Simonetti 2018; Selinske et al. 2023; Löfroth et al. 2024), the persistence and analytic traction of this oversimplified dichotomy highlight the need for deeper philosophical and ethical scrutiny. Different ways of how land use is conceptualized determine which solutions appear possible or desirable, which actors are held responsible, and which values can legitimately guide policy and practice (Leach et al. 2010; Scoones et al. 2015). There is also a need to better comprehend how to think and discuss the ethical ways of living together with the nonhuman world, the central question of cohabitability.

There is increasing attention in the scientific community to acknowledge that land use and biodiversity conservation are deeply ethical and value-laden topics rather than merely ecological, socio-technical, or administrative problems (e.g. Soulé 1985; Baard 2021; Pascual et al. 2021; Cortés-Capano et al. 2022). Research has reacted to this by the incorporation of more participatory, transdisciplinary, and cross-disciplinary knowledge production practices into biodiversity conservation and environmental management (e.g. Bennett et al. 2017; Turnhout et al. 2020; Massarella et al. 2021). However, despite these advances, explicit engagement with environmental ethics (i.e. the branch of applied philosophy concerned with normative reasoning about human–environment relations) remains sporadic in these fields (Baard 2021; Cortés-Capano et al. 2022). In this sense, ethics is often instrumentalized to rhetorically justify management choices or value framings rather than to interrogate the underlying assumptions about what constitutes a good, just, or responsible relation with the environment (Turner et al. 2014; Cortés-Capano et al. 2022). Strengthening the contribution of philosophical ethics to biodiversity conservation and environmental management thus is central to provide conceptual precision, theoretical frames, and argumentative depth for sustainability transitions.

Despite the centrality of human presence, activities, and land use impacts, to addressing human–nonhuman relations and related ethical challenges, environmental ethics has relatively rarely addressed these issues with an explicit land use vocabulary (including land sharing/sparing), comprehensive outlook, or integration to other land use and management topics. Discussions on agriculture, wilderness, restoration, or urban nature are often conducted in isolation and through different terminologies, making it challenging to trace how ethical positions have evolved and interrelate across land use contexts. As a result, knowledge about human–land relations in environmental ethics remains fragmented, limiting both the field’s internal coherence and its capacity to inform interdisciplinary debates in sustainability science.

This evokes a need to synthesise and interpret how environmental ethics has approached questions of land use, management, and human presence in diverse environments. This is increasingly important for informing sustainability transformations, where ethical framings shape what forms of human–nonhuman coexistence are imagined as possible, legitimate, or desirable. In this context, our main research question was: how has environmental ethics literature addressed the impacts and ethical evaluation of human presence and human–nonhuman coexistence in various environments?

To address it, we conducted a qualitative thematic literature review to identify and interpret the main narratives that have shaped ethical reasoning about human presence in nature and to explore how these narratives frame human–land relations, moral responsibility, values, and prospects for coexistence. Narratives constitute powerful analytical and normative devices that organise how complex socio-ecological realities are rendered intelligible, actionable, and politically meaningful (Bruner 1991; Scoones et al 2015). They frame problems, delimit the space of possible solutions, and distribute responsibilities by establishing particular logics of causation and moral agency (Leach et al. 2010). Simultaneously, narratives operate as sensemaking devices through which actors interpret uncertainty, negotiate values, and articulate collective solutions (Cortes-Capano et al., 2025). Environmental research has mobilised narrative approaches to address how specific framings gain authority and marginalize others (Roe 1994; Leach et al. 2010; Scoones et al. 2007). Such work aligns with genealogical orientations that trace how dominant storylines emerge, sediment, and evolve within broader epistemic, institutional, and socio-political configurations (McMichael 2009; Walker and Cooper 2011; Bless et al. 2023). Within this study, such a novel approach enabled us to complement traditional environmental-ethical analyses, which often centre on arguments about moral considerability or duties. This adds a historical perspective that treats the field as an evolving repertoire of narratives that frame human–nonhuman coexistence, structure ethical problem-spaces, and orient proposals for action (McMichael 2009; Bless et al. 2023). This makes our approach not only a descriptive exercise but also a critical and potentially transformative engagement with the plural ways environmental problems and responsibilities are constructed.

Our contributions are the following. First, our review creates new knowledge for interdisciplinary use by synthesising environmental ethics narratives around land use, management, and human–nonhuman relations. This advances cross-disciplinary learning and collaboration by introducing the main approaches, ethical propositions and theoretical underpinnings. Second, we identify key assumptions that ground different themes in the studied discussions, for which the narrative approach is especially valuable. This helps see both the strengths and challenges of different narratives, which also advances environmental ethics. Third, we reflect upon where environmental ethics could benefit from more interdisciplinary influence from disciplines that have for long been theorising and empirically studying land use and land management. Novel is also a genealogical perspective that looks at the co-evolvement of environmental ethical narratives as grounded in particular histories and contextually situated and shows their futures that are opening through the emerging discussions and contestations.

The following section introduces our methodology and research scope in detail. The results section describes the results organised under four main narratives identified from the literature and their key features, genealogy, tensions, and ethical implications that different narratives have on the human presence and actions. In Discussion, we reflect upon the implications and transformative potential of these findings for both sustainability science and environmental ethics.

## Methodology and methods

In order to portray the spectrum of themes that have emerged and become influential in environmental ethics, constituting different overarching narratives, we conducted a qualitative thematic review (Grant and Booth 2009). Qualitative reviews are interpretive in nature and seek to broaden understanding of a particular topic or phenomenon by identifying ‘themes’ or ‘constructs’ within a body of literature (Grant and Booth 2009). To ensure wide inclusion of relevant contributions, we conducted systematic searches in academic literature databases, complemented by purposive sampling. In May 2024, we applied a search string to three databases: Scopus,^1^ Web of Science,^2^ and Philosopher’s Index,^3^ including publications until May 2024. The search string included key terms such as landscape, land use, coexist, convivial and also urban, rural, agricultural, and environmental ethics (see Appendix S1 for search strings, coding key themes, thematically organized results, and the database of publications included in the review). It aimed at capturing academic English articles that would explicitly address environments with active human presence and/or form of land use (e.g. urban, agricultural, and forestry lands) in their title, abstract, or keywords. Since the broad-scoped topic has been extensively addressed by multiple academic fields of research, here we focus on contributions in and close to the field of environmental ethics. Because terms such as ‘land use’ and ‘land management’ are rarely employed explicitly in environmental ethics, we focused on publications addressing “human presence,” which is more commonly used in the field.

In the first screening we included peer-reviewed research articles (including possible literature reviews) to be later on assessed through manual classifications based on explicit inclusion and exclusion criteria. We excluded book chapters, book reviews, and commentaries (books were covered by complementary purposive sampling). After removing duplicates, the searches yielded 277 documents. We then read and assessed the titles, abstracts, and keywords to ensure inclusion of relevant articles following explicit inclusion and exclusion criteria. We considered an article to be relevant if it (i) explicitly discussed contexts of human land use (such as urban planning) or issues related to the human presence in various landscapes *and* (ii) did this with an environmental ethics perspective, which is characterized by interest in how human presence impacts on nonhuman nature and addresses such impacts from a philosophical ethics perspective. Animal ethics focused articles were included only if they also involved this general environmental-ethical perspective. Articles describing different aspects of coexistence yet lacking nonhuman perspective or engagement in the environmental ethics discussion, like purely empirical works, were not included. Finally, after manual sorting of the abstracts, we analysed the full text of the remaining documents for final eligibility resulting in 50 documents (see Appendix S1 for a full list, including purposive sampling references described below).

In addition, we complemented the database with purposive sampling aimed to capture influential and thematically central works shaping the field of environmental ethics that were not reached by database search. This yielded 36 additional pieces to our data (86 publications in total). Books for the analysis were chosen only via purposive sampling since creating sufficiently, yet not overly, inclusive database search criteria for books would have been practically impossible. Building on literature, our purposive sampling list was further refined through extensive collegial feedback, ensuring a peer-informed and extensive set of relevant sources. This list was narrowed down to a manageable size ranking the relevance, limiting the number of works from the same author, and then broadened with some new publications that emerged in the review-planning discussions. The guiding principle for determining the scope of purposive sampling was qualitative saturation at the level of analytical detail that our review was designed to encompass. Thus, the resulting list of purposive sampling works is not meant to suggest an exhaustive list of environmental ethics books that touch upon land use and management issues.

### Data analysis

The analysis followed a two-step analytical process combining thematic analysis (Braun and Clarke 2013) and narrative synthesis (Leach 2010; Scoones 2007). First, we conducted a qualitative thematic analysis to characterise how human presence, use, and ethical relations with landscapes were represented across studies (Braun and Clarke 2013). Themes represented analytic outputs actively produced through coding at the intersection of data, analytic process and inter-subjectivity (Byrne 2022). We followed an abductive reasoning orientation for coding and theme construction, involving a parallel engagement with empirical data (i.e. the review corpus) and with our existing, relevant theoretical understanding of environmental ethics literature in a broad sense (Timmermans and Tavory 2022; Cortés-Capano et al. 2025). Following Braun and Clarke (2013), after familiarising ourselves with the narratives, the authors conducted an iterative coding process aiming to identify and interpret patterns of meaning across the documents (i.e. relative core commonalities; Byrne 2022). These themes and their relations represented the foundation, to then conduct the narrative synthesis into coherent storylines.

Narrative synthesis aims to make sense of this complexity and emergence by tracing how ideas, ethical positions, and practices evolve, interact, and give rise to multiple interpretations (Grant and Booth 2009; Paré et al 2015). To structure the narrative synthesis, we developed an original analytical arc composed of 5 main elements: (i) origins, (ii) consolidation, (iii) contestations, (iv) implications, and (v) continuities and shifts. This heuristic arc was inspired by genealogical approaches to discourse and narratives (Tamboukou and Squire 2008). The origins trace the intellectual, geographical, and socio-political emergence of each narrative, situating its key assumptions in relation to broader scientific and policy debates. Consolidation captures how particular ethical orientations, ontological positions, and framings of human–nature relations become stabilised and reproduced through recurring concepts, metaphors, and methodological conventions. Contestations reveal the tensions, paradoxes, and internal pluralities that destabilise or enrich each narrative, highlighting how ethical positions are negotiated and developed. Implications present the normative, epistemic, and political consequences of these narratives, showing how they shape research agendas and practical justifications for certain environmental actions. Finally, continuities and shifts trace how these narratives evolve, hybridise, or give rise to emergent perspectives, enabling a generative and forward-looking interpretation. All narratives were iteratively discussed and reviewed by both authors to increase coherence, representativeness and credibility.

## Results

Our thematic analysis revealed four distinct narratives in environmental ethics regarding how human presence and human involvement with land or nonhuman nature are approached. These narratives were named as (1) Wilderness narrative, (2) Rural stewardship narrative, (3) Urban cohabitation narrative, and (4) Relational narrative.

Below, we present the summary of the key characteristics of these four narratives in Table 1 to provide the overview and help their comparison. Full thematic reports of results are available in Appendix S1, which also includes the list of analysed datasets to which references in Results are marked with prefix ‘D’ (for datapiece). After the table, we report our findings in a narrative form, discussing each of the four identified narratives in turn. A short terminological note is in place. When we speak in results of nonhuman–nature, we use ‘nature’ for convenience. However, in our own positionality, we consider that humans are part of nature, not separate from it.

**Table 1:** The four main narratives identified in the literature review and their focal characteristics

Narrative	Context	Spatial & temporal focus	Conceptions of human presence and impact	Ethical propositions & modes of engagement	Selected exemplary references
Wilderness	US preservationism; inherent value of nature to protect it	Large, remote biomes, large landscapes; historical deep-time baselines and also ‘atemporal’ features	Human absence valued; presence assumed disruptive; intervention only to restore ‘naturalness’	Non-interference, preservationist duties; land sparing; in rewilding debate managerial paradox	Taylor (1986); Katz (1993); Rolston (2001)
Rural stewardship	US agrarianism; farming practices and rural discussions	Regional or local spatial scale; short-term (decades) historical sensitivity to landscape changes	Human presence default state and can have positive or negative impacts; types and magnitude of involvement matter	Management for land health and ecological integrity; attentive concern; many roles and duties; land sharing and some sparing	Leopold (1949); Minteer (2008); Thompson (2010); Millstein (2024)
Urban cohabitation	Urbanization entering into ethics; environmental and social concerns partly incorporated; cross-disciplinary discussions	Urban city-level or metropolitan; small cities dismissed, peri-urban present sometimes; either atemporal or future-oriented	Human presence default state and can have positive or negative impacts; types and magnitude of involvement matter	Consequentialist and pragmatist tones except deontological urban animal ethics; promotion of social and environmental good together is ideal	Gunn (1998); Palmer (2003); Epting (2017); Dill (2022)
Relational narrative	Decolonial and/or non- Western; relational turn; critique of blind spots and problems in the Eurocentric scientific and philosophical project; indigenous and global South voices	Regional or territorial landscapes or significant habitats; strong historical awareness of colonial and Western trajectories	Human presence can have positive or negative impacts; the interconnectedness of human and more-than-human wellbeing emphasized and impacts regard also relations to land	Obligations regarding relations and their qualities; care and reciprocity; integration of environmental and social responsibilities; rejection of sharing / sparing dichotomy	Warren (2000); Rozzi et al. (2015); May (2018); Di Giminiani (2022)

### Wilderness narrative

The wilderness narrative represents one of the central birthplaces of contemporary environmental ethics, rooted in US preservationism and with close ties to the establishment of national parks as sanctuaries of pristine nature. US preservationism emerged in the late 19th and early twentieth centuries. As it was later consolidated in philosophical debates of the 1980s and 1990s, it reflected anxieties over modernization, industrial expansion, and the human imprint on landscapes once regarded as wild. This moral geography of purity was shaped by the preservationist ideal that nature’s highest value lies in its separation from human use, in its ‘pristineness’.

Over time, this ideal converged into a coherent ethical position establishing that nature possesses inherent, non-instrumental value, and human presence, either physical or indirect, inevitably degrades it (D19, D63). The narrative’s spatial focus is rooted in large wilderness areas, ecosystems, national parks, and biomes, where ecological integrity is associated with human absence or very low density of any (and preferably temporary) human presence (D63). Temporally, the narrative is often grounded on both deep ecological time and historical baselines preceding industrial disturbances. Its normative orientation is largely deontological: moral duties of non-interference, respect for autonomy, and preservation of non-artefactual nature should define the human–nature relationship (D74, D34). In practice, these commitments translate into a ‘land sparing’ ethic with the obligation to set aside extensive tracts of land as strictly protected to minimize human presence and impact. Yet this same ethic gives rise to a managerial paradox: preserving natural autonomy might in the world of global environmental changes often require active human intervention. The removal of dykes, canals, and other artefacts to restore ‘natural’ processes exemplifies this contradiction, revealing that human withdrawal must sometimes be enacted through purposeful presence and action.

As debates evolved, internal tensions in the narrative emerged. Some critics questioned the essentialist assumption that naturalness could not be restored once lost, and whether human involvement always undermines ecological values. The rewilding narrative, particularly influential in Europe, reinterprets intervention as potentially generative since human actions might be necessary to enable or restore nature’s autonomy, facilitating processes of ecological self-organization (D44, D37). This challenges the strict dualism between nature and culture that defines the original wilderness ideal. Despite this, the wilderness narrative remains dominated by universalist and abstract framings that keep humans and nature separate. Humans also remain depicted as a singular, undifferentiated species; exceptions occasionally acknowledge Indigenous peoples or conservation practitioners. Nonhuman nature is treated in a holistic and generalizing way, emphasizing ecosystems or landscapes rather than specific species or individual sentient or non-sentient beings, with some exceptions around predator–prey discussions as part of discussions concerning wildlife management (D18, D5).

Ethically, this narrative sustains preservationist commitments as the primary concern for environmental ethics. An extreme manifestation of this is the delimiting of the scope of environmental ethics theory to concern ‘a system of moral principles by which human treatment of *natural ecosystems and their wild communities of life* ought to be guided’ (D74, D9). In less extreme variants, environmental ethics can cover a broader scope yet remains fully disconnected from social justice concerns. It reinforces a vision of moral responsibility as withdrawal rather than engagement: the right human act is to retreat and protect.

Originally, the distinctiveness and strength of the wilderness narrative and its ethical postulates were probably very influential in making environmental ethics a field with its own identity and mission. The strong emphasis on the inherent value of nonhuman nature made the normative project distinct from almost anything that had been done in ethics before (even if philosophy's long history had witnessed a few authors who had given some consideration to the inherent value of nonhuman nature or caring for it as a general obligation or virtue, regardless of its usefulness for human purposes). The wilderness narrative endures as a powerful symbolic and moral force in environmental thought, continuing to inspire protectionist and ‘sparing’ strategies. However, this distinctiveness also prevented the building of bridges to many other discussions. Hence, recent shifts, particularly the rise of rewilding, signal a more plural reorientation of wilderness thought (D5, D18). Rather than abandoning the value of autonomy, these emerging framings reimagine wilderness as a co-produced condition, achieved through negotiated and temporally open relationships between human and nonhuman actors (D60, D37). The wilderness narrative thus persists, but in more porous forms, often beyond untouched ideals, to embrace a living tension between care and restraint.

### Rural stewardship

The rural stewardship narrative traces its intellectual roots to the Leopoldian strand (D38, D10, D11) of environmental thought (although Leopold’s works also nurtured the wilderness debate due to their richness that left room for diverse interpretations) and to agrarian values that foreground care, place, and practice (D77). Emerging within an explicitly rural context, it orients ethical attention to biotic communities often shaped by farming, grazing, and small-scale land management rather than to remote ‘pristine’ wilderness. Unlike the wilderness narrative, which privileges large biomes and deep-time scale baselines, rural stewardship is rooted in the notion of the rural and the size of local ecosystems: areas where everyday lives are lived and where people work the land (D12, D44, D38, D31). Ecosystems or biotic communities (that some also call land communities to include abiotic elements; D43) are usually conceived as historical with layered short-term histories, often measured in decades or a few centuries. From the context and spatio-temporal scale follows the central perception that humans are not seen as external or as threats to landscapes but embedded members of biotic communities whose practices materially and symbolically co-constitute landscapes (D43). The narrative therefore builds on values and commitments to stewardship, citizenship, and professional responsibility. This has been referred to by some authors as part of a ‘third-way’ environmentalism (D44), not that different from pragmatism, which rejects and tries to go beyond the binaries juxtaposing nature/culture or posing anthropocentrism and non-anthropocentrism as dichotomic categories.

Over time this orientation consolidated into a broad yet coherent ethical position. Its core propositions hold that land may be given holistic ethical regard without foreclosing productive use (D38, D41, D28, D48). Soil integrity, biodiversity, and biotic or ecosystem or land health are viewed as central normative concerns. Ethical cohabitation is perceived as enacted through care, skill, and civic and professional virtues and engagement with nature that follows these principles. Spatially and temporally, the narrative focuses are local–regional and practice-centred, sensitive to place histories and to the particular cycles and rhythms of soils, crops, and herds (D32, D48). Epistemically the rural stewardship narrative privileges both ecological science (e.g. soil science, ecology) and embodied, practice-based knowledge of farmers or other professionals who know and work the land. Linked to the ecological and professional tones, some contributions particularize nonhuman life forms to a significant extent (D38), but differentiation often focuses on classes, such as different levels in food chains, and especially attention to individual beings is rare. The distinction between wild and domesticated remains strong. Ethically, the rural stewardship narrative is often plural or pragmatist as deontological duties of care are considered together with consequentialist attention to impacts, virtue ethics of humility and stewardship, and relational ethics that centre responsibilities within local relations and ecologies (D77, D78). This could also be interpreted as the lack of a cohesive ethical foundation. The ‘farmer as conservationist’ ideal (D38) exemplifies the consolidated importance of professional practice, that, when guided by stewardship, can become a means for responsible cohabitation.

Internal debates both enrich and complicate discourses under this narrative. The first line of disagreement concerns the ethically appropriate degrees and modes of human agency. Some accounts emphasise non-hierarchical membership in biotic communities, others allow for modest hierarchy based on technically informed ‘managerial stewardship’ that, to some, leans too much on control. The narrative therefore negotiates the land sharing—sparing divides. On one hand, it leans towards sharing, rooted on the notion that managed disturbance, and traditional biotope grazing, rotational tillage, or alike practices can enhance community-level regeneration capacities. On the other, it manifests worries about such management going ‘too far’: there would also be some sparing or ‘letting-them-be’ to do. Restoration discussions occupy a particular contested space. Nevertheless, responsibly conducted restorative practices are often framed positively as acts of care that can repair soils and processes, contrasting with wilderness claims that restored systems remain artefactual. Religiously rooted arguments and discussions (e.g. stewardship rooted in Judeo-Christian dominion) appear sporadically, often provoking further debate about the grounds of responsibility. The “professionalist ethos” (D12, D38, D43) can also generate tensions by privileging farmer knowledge as the authoritative perspective. This emphasis can unintentionally sideline other rural actors, particularly those already facing structural disadvantage, such as landless workers, Indigenous communities, and non-farmer rural dwellers. By doing so, it risks valorising already-powerful practices and identities while overlooking marginalised voices and knowledges. While some of the ‘third-way environmentalism’ approach encompasses both urban and rural considerations, there are some notably anti-urban tones within the rural narrative (see also De-Shalit 1996; 2003). Such tones portray rural life as superior or better, or urban life as somehow degraded (D12). De-Shalit suggests that this is echoing the anti-urban attitudes of a Judeo-Christian tradition; this is in line with our findings where the rural stewardship narrative has a strand with religious ‘pastoral’ references or even underpinnings (albeit it should not be equated with a religious approach).

The narrative entails concrete moral and policy implications. It legitimizes an integrative land ethic where human wellbeing and land health are interdependent and can be mutually reinforcing. It also supports policy measures that promote agroecological practices, skills training, and place-based stewardship actions and institutions such as community-led habitat restoration initiatives, participatory monitoring and neighbourhood associations. Its attention to soil health and ecosystem processes reframes conservation as an active, ongoing practice rather than spatial exclusion around nature reserves. At the same time, by not sharply separating social from environmental concerns, the narrative opens space for civic and political virtues such as environmental citizenship, local governance, and collective responsibility, to have a central role informing and judging environmental action. However, the emphasis on local practices also raises questions about scale: how do regional stewardship models respond to market pressures, large-scale industrial agriculture, or planetary processes such as climate change?

The rural stewardship narrative continues to contribute both to debates in environmental ethics and to environmental action through its valuation of land integrity, practice-based knowledge, and stewardship responsibilities. Shifts appear as the narrative extends its temporal and spatial horizons; some authors scale stewardship even toward planetary thinking (‘thinking like a planet’, as phrased by Callicott 2013; ‘earth stewardship’, as developed by Chapin III et al. 2022). Engagement with critical strands that challenge technocratic modernization and productivism is ongoing yet also comes with more varied views on the proper role and use of technology and modernization. Rearticulations also emphasize plural ethics, the co-production of local ecological knowledge between scientists and practitioners, and the possibility that some levels of respectful control and restorative interventions can coexist with humility and care, as part of biotic communities. Thus, the rural stewardship narrative remains both rooted and adaptive, and often (albeit not always) reframes human presence in landscapes beyond dualisms (D31, D78), to be associated with a morally charged set of practices through which flourishing soils, species, and communities are possible.

### Urban cohabitation narrative

The urban cohabitation narrative emerges from a long-standing gap in environmental ethics: the sidelining of cities in favour of wilderness areas. Only quite recently, the empty space for such narrative started to become addressed by a gradual disciplinary turn toward urban socio-ecological systems and problems. Early critiques of an ‘urban blind spot’ (Light 2001) and calls to bring cities into ethical inquiry catalyzed a more concerted interest from the 2010s onward. Its origin is multidisciplinary as environmental ethics for urban environments draws on political ecology, human geography, urban planning, and environmental justice research, while the connections are sporadic and not systematic. It centres the distinctive materialities of city life, including built infrastructure, transport, lighting, and human practices, to foreground human–nonhuman entanglements unique to urban settings.

As the narrative consolidated, core assumptions and propositions became increasingly more visible. The narrative assumes human presence as self-evident and constitutive of place, leading to conceptions of cities as built environments in which ‘nature-in-city’ matters ethically. Contrasting to highly biodiversity-oriented considerations in the wilderness and rural narratives, the urban narrative proposes that ordinary, urban nature, trees, pigeons, insects, and even streets and urban metabolisms bear significance (this does not imply that all would hold moral worth in themselves) (D25, D46). Human impacts are not categorically harmful but contingent on degree, including form and impact of modification (D17, D21, D51). Spatially, the scale is typically the city or metropolitan region. Temporally, many treatments are ahistorical or short-term, though some works trace decades-long urban trajectories and others focus on future adaptation challenges. Normatively, the urban narrative has plural and consequentialist tendencies: contributions often focus on the impacts of actions (except for animal-oriented approaches, as we note later), yet rarely assume a single good as determining what is right or good. In addition, urban animal ethics introduces deontological duties and care relationalities mainly regarding sentient urban animals (D51, D46). Weakly anthropocentric logics are common. While some propositions suggest that any urban ethic must be at least weakly anthropocentric (D21), the centrism debate has not entered urban environmental ethics. Relatively technology-neutral stances are common while also often embrace nature-based solutions.

Also in this narrative, internal contestations enrich and fragment the debate. One such division separates a general urban environmental ethics, concerned with ‘nature’, air quality, and city metabolism (D21), from a distinct urban animal ethics that foregrounds entitlements, care, and hospitality toward sentient cohabitants (D46, D51, D42). Although their implications overlap, for example, when addressing the impacts of climate change, these sub-narratives rarely engage in direct dialogue. Tensions exist regarding *what or who* in urban nonhuman nature require ethical attention: nonhuman nature is particularized yet in unsystematic and sporadic ways, discussion varying from individual beings to species living a state of being neither fully wild nor fully domestic, to large animal classes and ecosystems. Methodologically, critiques point to an ahistorical tendency and ‘overreliance’ on environmental sciences and sustainability discourse, even as some studies incorporate marginalized urban experiences and everyday experiences of urban residents. Normatively, debates are still mixed about the larger role of cities in environmental ethics: whether cities should be framed as new sanctuaries for nature (revalorizing urban conservation) (D13) or seen as engines of habitat loss where attention is just required to small-scale promotion of nonhuman good. Nevertheless, the inevitable sharedness of urban environments makes the perspective focused on the ethical differentiation between different forms of land sharing rather than engaged in the sharing/sparing debate. Other unsettled questions include whether nonhuman differentiation should prioritize species, individuals, or abiotic urban elements; and how to reconcile technology-based interventions with relational ethics of coexistence and contested views about how technologized cities are ‘good cities’.

The narrative broadly supports integrative urban design, including green corridors, native planting, lighting reforms, and planning that attends to multispecies mobilities (D17, D46). In addition, it often at least implicitly supports governance that differentiates human responsibilities, including those of planners, communities and citizens. It reframes conservation as multi-scalar and practice-oriented, as protecting biodiversity in cities involves social justice, everyday ethics, and attention to metabolic flows (e.g. pollution, circadian disruption, climate impacts). In animal-focused strands, duties of care and hospitality justify interventions to assist (but also to maintain respectful distance from) urban wildlife (D51, D42). This orientation makes the narrative hospitable to technology and nature-based solutions, but it also demands context-sensitive evaluation that foregrounds social equity and lived experience.

Continuities include the narrative’s persistent move away from wilderness dualisms and its sustained claim that ‘ordinary’ urban nature matters ethically too. Shifts are observable in the widening of geographic representation and interdisciplinarity and in increasing discussion about species-level and individual animal concerns alongside broader ecological qualities. Moreover, future-oriented works that engage adaptation and long-term urban trajectories are starting to bring intergenerational ethics influence into the urban context. The narrative is adaptive as it retains a core integrative proposition, that human–nonhuman cohabitation in cities can and should be ethically guided, while pluralising methods, scales, and normative positions. Urban cohabitation reframes human presence beyond simple disturbance to be an ongoing set of relations that require ethical approaches, civic imaginaries, and differentiated responsibilities to secure just and biodiverse urban futures.

### Relational narrative

The relational narrative finds its roots in intellectual and ethical traditions that largely contest Eurocentric universalism or dualisms. Direct contestations of the Eurocentric features are most visible in early ecofeminist contributions and in Indigenous and Global South literature, while Asian literature rarely involves similar criticism towards the Eurocentric positions and has, rather, developed as a distinct discourse. Direct contestations focus on criticising the wilderness narrative (D24), for reproducing colonial power relations, disregarding the social plights and environmental realities, and dismissing how ‘setting aside of wilderness’ worsens social inequalities in ways that environmentalists traditionally neglect. When humans exercise colonial power, they also often treat the nonhuman world (or nonhuman inhabitants) badly and exercise ecological imperialism.

Despite the wide-branching roots of relational thought, this narrative has remained marginal in environmental ethics until it started to strengthen during the 2010s (according to our observations). Its renewed momentum arises from two converging forces: the resurgence of decolonial critique and the revival of feminist ethics of care (D59, D85). These forces are deeply situated in political and geographic contexts, Indigenous territories, colonial frontiers, and regions of the Global South. In these geographies, ethical and political thought and life have long been animated by critiques of imperial histories, extractivist development, and epistemic domination from Eurocentric, rationalist perspectives (D83). From the critique set by the forces follows a central tenant: humans and nonhumans are constitutively entangled, due to which the Western binaries of nature/culture, reason/emotion, and human/nonhuman are inadequate and complicit in systems underpinning the environmental crisis (D59, D83, D66). Relational ethics begins from lived interdependence and the recognition that caring for a world requires learning its languages, histories, and capacities to respond.

As this literature evolves, a distinctive set of propositions emerges. Land, landscape, and life are understood relationally, as kin, commons, or co-constituted fields of belonging, rather than as external objects, aesthetical background or containers of human activities (D15, D39). Human presence is seen as inherent to the way the land is and becomes positive or negative for nonhumans only when taking its shape through the various practices and enacted in human–land or human–world relations. Ethical attention highlights reciprocity, cohabitation, and situated responsibilities (D52) over universalized rights detached from context. In spatial terms, the relational narrative grounds ethics in specific territories and ecologies such as local forests, watersheds and places which hold both material and symbolic significance. Temporally, it foregrounds historical depth and asserts that present injustices are understandable only through acknowledging and challenging colonial and extractivist trajectories. Knowledge pluralism is central as scientific ecology, embodied practice, oral traditions, and other ways of knowing mutually inform and sustain interdependence (D32). Within this framework, care is defined not as a one-directional act of benevolence but as a mutual articulation of vitality (D20). For example, drawing from indigenous understandings of agriculture and forestry, care entails reflexive awareness of its limits, of the recalcitrance of soils, forests, and waters to human intentions, and attentiveness to the conditions that allow nonhumans to affect and enchant humans in return (D15). Such recognition transforms care from management to relation, requiring humility before the agency of others. Universalism is rejected both epistemically and ethically as relational narratives sustain that ethical claims must be anchored in situated histories of cohabitation, privilege and dispossession. At the level of language, there are calls for a grammar of animacy that captures an essential decolonial insight: that speaking with, rather than about, other beings (when addressing water, soil, or wolf as animate kin, for example) reconfigures the very terms of coexistence and resists the colonial grammar that renders the world passive and prone to domination (D57, D85).

Internal differences and contestations animate the pluralism of the relational narrative. On one side, there are decolonial-ecofeminist voices, mainly rooted in Latin American, Indigenous, and Global South scholarship, which emphasise historicity, power, and call environmental ethics to contribute to decolonial praxis. On another side, Asian relational traditions foreground nondualist cosmologies and embodied harmony but engage less directly with colonial critiques. Within the relational narrative, tensions unfold in the relations between individual and collective: for example, how to integrate relational ontologies with individual rights, or recognising that caring for a whole living community may require short-term harm to some beings (e.g., periodic burning to conserve grasslands), while still being grounded in an ethics of reciprocity and regeneration. Some warn that care discourses risk romanticising harmony without addressing structural domination, while others see relationality as a radical ground for transforming political economies. Additionally, one central source of contestation is also whether and how much decolonial works should engage with the theoretical and conceptual products of the mainstream environmental ethical thought that is perceived as built on colonial heritage.

Normatively and politically, the relational narrative reframes obligations as practices of coexistence and response. Ethical action includes the cultivation of reciprocal relations, storytelling and defence of access to land against dispossession (D59). These oppose technical management and external stewardship. Policy implications include strong decentralization of decision-making, the recognition of territorial rights, Indigenous sovereignty, plural governance, and the integration of social justice and ecology. Conservation is reimagined as a practice of relations rather than separation: protected areas become living territories of interdependence where human and more-than-human agency are co-constitutive in mutually beneficial ways. The narrative also aligns environmental transformation with struggles against gender, racial, and colonial oppression, asserting that there is no ecological repair without social repair. Beyond indigeneity, newcomers to a place can cultivate ethical belonging through linguistic, affective, and legal transformations that respect Indigenous sovereignty and sustain relations of care without appropriation (D85).

Continuities in the relational narrative include enduring commitments to plural epistemologies and ontologies, and the ethical centrality of care and reciprocity. Recent shifts reveal an expansion of dialogue and recognition as decolonial, and ecofeminist frameworks move from the margins into institutional and policy arenas. The narrative is thus both continuous and transformative; it remains centred around the critique of Eurocentric dualisms while advancing the bridging of ontological pluralism, justice, and care. It calls for humans to inhabit the world from within its living relations, where ethical life emerges through reciprocity and response rather than through stewardship or management. Seen genealogically, the relational narrative offers not merely an alternative ethics but a very different worldview (or mode of worlding), one in which cohabitability is inseparable from decolonial struggles and the ongoing practice of becoming response-able to others.

## Discussion

Our review identified four main narratives within environmental ethics that take a different viewpoint to the human presence in various environments: wilderness, rural stewardship, urban cohabitation, and relational narrative. These narratives are distinct yet not sharply separated, as they have overlapping areas (even a single work can sometimes reflect different narratives in different parts) and dialogical exchange that is influential to their argumentation and conceptual development. The wilderness narrative marks one of the central birthplaces of contemporary environmental ethics and emerged in the US preservationism context. With emphasis on the values of naturalness and wilderness, the wilderness narrative upholds strong dualism between cultural and natural and argues for the minimized human presence in natural environments (maximal land sparing approach) to protect their inherent values that human activities can only eradicate. An exception to this can be seen in the recent discussions on rewilding that renew the wilderness narrative by viewing wilderness as a condition that can also be co-produced by human and nonhuman actors, in which cases the ‘wild’ and ‘human’ do not appear anymore antithetical. Rural stewardship and urban cohabitation narratives had very different contextual settings as they focused on environments where humans are by definition present. These narratives maintain that human impact on nonhuman nature can be either positive or negative and often embrace the sharing perspective to land use and management. The rural narrative highlights human–land relations, ecological interconnectedness, and professional roles (of farmers, forest managers, etc.), and the combination of land sharing and sparing. Attention shifts from grand wilderness to more local landscapes where responsible farmers can act as conservationists and stewards in their biotic communities, as people-in-nature. Urban cohabitation narrative, instead, flips the viewpoint into thinking about nature-in-city and how the pluralities of coexistence in both human and nonhuman diversity can be made possible. The question of cohabitability in diversity makes the land use perspective focused on land sharing, and for the same reason, social and environmental questions join in the urban narrative more often than in the wilderness and rural narratives. Urban narrative also emphasizes that ‘ordinary nature matters’ and calls for more attention to human–nonhuman encounters as spaces of both care and tension. The fourth narrative that we identified, the relational narrative, can be read as the alternative or critical voice to what had become mainstream in environmental ethics. It also rejects dichotomies such as those used in the sharing/sparing debate. While relationality and increased attention to relations marks this narrative strongly, the decolonial–ecofeminist strands and non-Western strands within it engage in different discussions. The integration of social and environmental ethical issues is inseparable in the decolonial, and ecofeminist discussions of this narrative and environmental ethics becomes dual-tasked with addressing both social and ecological histories of domination simultaneously.

### Working with environmental ethics to advance transformative sustainability science

Together, the identified narratives illustrate a sustained broadening of ethical focus and consideration. Despite overlaps and some exceptions, getting a somewhat chronological overview of the influence of various narratives in the field’s development is possible: the wilderness narratives began the story, followed by the rural stewardship, then the urban cohabitation, and finally the relational narrative. This development marked the expansion of ethical horizons from notions of separation toward coexistence, from wilderness to interdependence, and from ideals of purity in nature and minimization of human–nonhuman relations to the plurality of possible and appropriate relations. The relational narrative, whose strengthening position is chronologically the most recent development, has highlighted especially the plurality and the different ways of looking at relations. Interestingly, each narrative expresses a distinct grammar of human–nature relations: intactness and autonomy define the wilderness ideal, care anchors rural stewardship, coexistence characterises urban ethics, and reciprocity underpins relational thought. Across these variations, environmental ethics branches further from envisioning nature as a realm apart to conceiving it as co-constitutive and co-generative with human life and practice. However, this shift remains ethically unsettled and continuously evolving as tensions between autonomy and cohabitation, and restraint and involvement, persist. These tensions are constitutive of a plurality of ethical positions rather than resolvable (Cortés-Capano et al. 2022) and are, perhaps, better viewed as also reflecting the plurality of human coexistence with other lifeforms and in different landscapes.

Temporally, the narratives engage diverse registers of time. This includes the wilderness ethic, invoking deep ecological and geological timescales as baselines for purity (Cronon 1995). Rural stewardship works within generational (yet also seasonal) rhythms of agricultural and ecological succession (Barry and Smith 2008). Urban cohabitation commonly navigates short-term and future-oriented technological and social shifts (Epting 2017). Relational narratives foreground layered historical timelines that often include ongoing colonial dispossessions and decolonial responses (Drenthen 2018).

Spatially, narratives map distinct moral geographies (Smith 2000), understood here as the spatial organization and contestation of ethical values, responsibilities, and forms of moral concern across places and scales. Wilderness narratives often emphasise large or iconic landscapes and species while grounding ethical concern in particular places and encounters (e.g. specific parks, Cronon 1995). Beyond this, attention in different narratives extends to managed rural mosaics and more intensively and intimately inhabited urban territories, with occasional attention to very detailed organismal encounters (e.g., Michelfelder 2018), and to territorially situated Indigenous peoples’ lands where the importance of particular places is grounded in long-standing relations to specific places (Di Giminiani 2022). Together, these narratives illustrate how ethical attention is spatially organized, highlighting certain relations over others beyond simple distinctions between large and small absolute scales (Smith 2000; Massey 2005). The expansion of spatial perspectives is best understood as cumulative, where environmental ethics continues to encompass expansive viewpoints, including ‘thinking like a planet’ (Callicott 2013). For example, large-scale framings may centre around global responsibility or species-level concerns, while more situated framings make visible everyday relations and locally embedded harm and practices of care that might otherwise be overlooked (Plumwood 1993; Puig de la Bellacasa 2017).

While the expansion of temporal and spatial perspectival scales diversifies the capacity of environmental ethics to address human land use and human–nature relations with greater detail, our review found very little discussion about the systematisation of the use and dynamics of scales within environmental ethics. This calls for further attention in the field, as choices of scale significantly influence what becomes ethically visible such as particular actors, harms, or responsibilities, and what remains marginalized in different ethical discussions.

Each narrative has its own strengths and weaknesses. The strength of wilderness narrative lies in the theoretically foundational work and strong establishment in affirming nonhuman nature’s inherent value. It can lead to strongly motivated and passionate environmental action. Its weakness lies in constraining possibilities for relational coexistence and restricting the possibility of finding any positive role for humans in nature (Hettinger 2002; Ladkin 2005) and possible prohibition of actions that would benefit nonhumans themselves by, for example, improving ecosystem health (Thompson 2010). Rural stewardship and urban cohabitation narratives affirm the possibility for the positive role of humans in respectful and nonhuman-benefitting coexistence. On the other hand, they have a tendency to still uphold the urban vs. rural dichotomies (De-Shalit 1996), which may prevent the adoption of more holistic approaches to land use and planning. Both narratives also demonstrate tensions between the professional practitioner ethos and the inclusion of more local, participatory engagements. The strength of the relational approach lies in foregrounding the moral significance of situated human–nonhuman interactions and the historical, cultural, and ecological contexts (Whyte 2018; Pape 2024), which enable environmental ethics to address place-based attachments, socio-ecological entanglements and the legacy of injustices shaping current landscapes (Whyte 2018). Its weakness lies in its conceptual openness, which can make it difficult to evaluate competing relational claims or derive clear guidance for actions, risking either indeterminacy or ethical relativism. Dialogical exchange between narratives has pressed for addressing the weaknesses, and even provided ways to overcome them (e.g. influence of relational approaches to rewilding sub-narrative). Narratives also demonstrate how a fixed stance on the sharing/sparing debate (wilderness narrative, except for its recent strands) can create blindness to issues that more flexible approaches with multiple perspectives to land use can help make visible.

### A pragmatist and multiperspectival engagement with environmental ethics

As our results reveal, each ethical narrative emphasises distinct grammars of relations such as autonomy, care, coexistence and reciprocity. These imply partly distinct views on how the ethically responsible human action is framed. In the interdisciplinary and practical application of environmental ethics, a multiperspectival approach grounded in pragmatist philosophy might hold significant transformative potential by embracing the complexity and pluralism inherent in contemporary socio-ecological challenges (IPBES 2024). By engaging simultaneously with diverse narratives, a multiperspectival approach can foster a richer, more nuanced understanding of environmental dilemmas (Gardiner 2011; Mouffe 2013). This kind of strategy has already been embraced by many scholars beyond philosophy but also some philosophers, of which Paul B. Thompson’s works on agrarian ethics, Ben Minteer’s third-way environmentalism, Bryan G. Norton’s adaptive management, and Ricardo Rozzi’s biocultural ethic might be considered as examples. Much like multiperspectival democracy theories that encourage dialogue and negotiation among diverse political visions (Asenbaum 2022), engagements with environmental ethics would benefit from a pluralist orientation by cultivating ethical reflexivity that is responsive to varied temporalities, spatialities, and historical contexts (O'Neill et al. 2008; Dedeurwaerdere and Six 2016; Cortés-Capano et al. 2022). They can aim to agree about convergent goals and action despite different worldviews and epistemologies as starting points (cf. Naess 1989; Norton 2005). This plural engagement encourages researchers and practitioners to immerse in, or at least integrate, multiple ‘realities’ and ethical standpoints, enabling innovative synthesis without erasing difference. This shifts the focus from seeking definitive answers to indeterminate problems (e.g., climate change, biodiversity loss), which can still be seen as constitutive to basic research in environmental ethics, to embracing generative tensions as productive sites for ethical and political action. Thus, a multiperspectival pragmatist framework can catalyse new modes of governance and sustainability transitions characterised by adaptive, inclusive, and justice-sensitive responses in the world of interlinked environmental and social crises. This approach applies environmental ethics and its selection of sensemaking tools into the dynamic arena of practical transformation, where plural voices co-construct resilient and equitable futures (Puig de la Bellacasa 2017).

### Responsibilities to whom? On portraying nonhumans

One thing that surprised us in review findings was the invisibility of nonhuman diversity within the visibility of nature. In other words, while all narratives reflected the core idea of environmental ethics by their attention to *nonhuman* nature, this nonhuman surprisingly often remained generalized and undifferentiated; exceptions to this were found, but not in significant amounts, and mostly in the urban cohabitation narrative. Oftentimes, the argument was made for respect or care for, or obligation to, either ‘nature in general’ or to individuals, species, ecosystems, or relations. Where are the nonhumans in all of their diversity in environmental ethics?

We suggest that environmental ethics would benefit from greater differentiation of the nonhuman world and that cross-disciplinary engagements are particularly valuable for this purpose, as Aldo Leopold’s land ethic (Leopold 1949) and Holmes Rolston III’s discussion on duties to endangered species (Rolston 1985) demonstrate. First, calls for respecting nature might appear artificial and impactless without differentiation: it is doubtful whether respect for nature can be realised without sufficient particularisation to acknowledge the differential needs and vulnerabilities in the given context (Kortetmäki et al. 2023). Second, better connecting environmental ethics to real-world challenges requires improved particularisation as normative positions often remain so abstracted that they can be ‘translated’ to support almost opposite kinds of ad-hoc solutions and to give ex-post justification, rather than ex-ante guidance. Third, the common assumption that duties to nonhumans match with, and only with, a particular level-of-life (as debates between biocentric individualists and ecosystem-oriented holists suggest) dismisses the diversity within these levels. Some of the organismal entities differ enormously from each other and much more than some organismal entities differ from certain non-individual entities (for example, a beetle or a whale differ more from each other than an Amazonian tree, hosting a really diverse set of species, differs from the forest that the tree is part of). Finally, if transformation calls for the diversification of voices in the ethical and political arenas, it is important that environmental ethics claims for the diversity of nonhuman voices (regardless of how they are represented or listened to), to properly recognise the diversity of both human and nonhuman voices in future-constructing arenas.

### Limitations of the study

We acknowledge that this study has several limitations. The search was limited to the English language, which excludes meaningful pieces published in other languages. In addition, despite our reflexive construction of the sampling strategy, including search strings, different databases and purposive sample, we are aware that it may not have captured all existing works on the topic within the field. For example, it must be noted that our search strings helped incorporate those ‘alternative works’ that directly contest Western environmental-ethical thought, but the ‘non-contesting alternatives’, such as Chinese, Indian and Japanese environmental ethics, are totally underrepresented in our analysis. We may also have dismissed relevant literature on some specific land use types, such as forestry and marine systems. We mitigate these limitations by making our procedures explicit, providing the full corpus list for reproducibility, and situating the narratives as interpretive constructs intended to illuminate rather than exhaust the ethical landscape. Our intention was not to represent the full diversity of perspectives within environmental ethics, but to articulate the main narrative formations in a genealogically informed way that surfaces their assumptions, highlights their ongoing openness, and contributes novel analytical tools to current conversations in environmental ethics and sustainability science and practice. A genuinely global review on the matter would be a very valuable addition to expand our work and reflect upon its findings in the future.

## Conclusion

What environmental ethics has to say about land use and management is highly important for transformative research and practice on land use and management and biodiversity conservation. This literature review on the human presence in environmental ethics sought to give a good overview on how the human–nature relations and human presence and impact appear in different strands of environmental ethics. The aim was to foster cross-disciplinary fertilization of concepts, arguments, and theoretical frameworks in topics that are permeated by ethical and value issues. Another aim was to increase the self-understanding of environmental ethics itself with a broader, genealogical overview of how its human–land and human-involvement related discussions have evolved.

Different narratives have their own appeal, strengths, and weaknesses and our study showed that it is neither possible nor productive to claim the superiority of one over the others. Each of the narratives has theoretical and conceptual elements that can be valuable to certain contexts or to the mapping of different ethical concerns with stakeholders and participatory approaches to biodiversity conservation and land use and management. We recommend increasing the cross-disciplinary incorporation of environmental ethical elements into research on biodiversity issues and human–land relations but with sufficient awareness of their theoretical and normative commitments and implications, which our review seeks to make visible. This helps transformative research in future to improve problem framing, in order to create better maps for determining what kinds of solutions are sought to address the human and nonhuman coexistence and to improve the cohabitability of different landscapes.

## Supplementary Information

Below is the link to the electronic supplementary material.

Supplementary file1 (PDF 211 KB)

## Footnotes

1
www.scopus.com.

2
www.webofknowledge.com.

3
https://philindex.org/.
