# Supplementary material for: From wilderness to cohabitation: Literature review on human presence in environmental ethics
Source: Ambio. 2026 Apr 29;55(10):2395–409. doi: 10.1007/s13280-026-02377-z (PMC13554035; doi:10.1007/s13280-026-02377-z)
Supplement: Supplementary file 1 [file 13280_2026_2377_MOESM1_ESM.pdf]

Supplementary information S1

Supplementary Information: This Supplementary Information has not been peer reviewed.

Title: From wilderness to cohabitation: Literature review on human presence in environmental ethics

Contents

- Search strings for the literature review
- Thematic results for the four main narratives
- The database of publications included in the review

Search strings

Scopus

((TITLE-ABS-KEY ("landscape*") OR (TITLE-ABS-KEY ("land use*")) OR (TITLE-ABS-KEY ("coexist*")) OR (TITLE-ABS-KEY ("convivial*")) OR (TITLE-ABS-KEY ("cohabit*")) OR (TITLE-ABS-KEY ("coinhabit*")) OR (TITLE-ABS-KEY (urban*)) OR (TITLE-ABS-KEY (agricultur*)) OR (TITLE-ABS-KEY (rural*)))) AND (TITLE-ABS-KEY ("environmental ethic*"))

Web of Science

(TS=(landscape* OR “land use” OR coexistence OR convivial* OR cohabit* OR urban OR rural OR agricultur*)) AND TS=("environmental ethic*")

Philosopher’s index

Philosopher’s index (Ebsco) has much more limited possibility to adjust the syntax and there is no option to search Field ‘topic’ (title, abstract, keywords included) as a whole.

(landscape* OR “land use” OR coexistence OR convivial* OR cohabit* OR urban OR rural OR agricultur*) AND "environmental ethic*" from All text

Codebook for the key themes

Initially, the codebook consisted of a list of key themes and subthemes, a label and definition, and instructive questions to guide how to use and categorize findings under themes in the corpus including exclusions and examples (MacQueen et al. 2008). As the familiarization with the material deepened, the original codebook was complemented with two additional key themes and their subthemes to enrich findings into directions that were found as providing important additional value.

The key themes of codes and examples of their subthemes are presented in Table S2.1.

Table S2.1 Key themes and subtheme examples that guided coding.

**Key themes and their short explanations**	**Subtheme examples (and their subcategories if applicable)**	**Example from coding**
Context:<br><br>describes how contextual factors are addressed or contextual positioning done	Urban<br><br>Rural<br><br>Agrarian<br><br>Western environmental thought<br><br>US conservationism<br><br><br>+spatial or temporal focus	"…urban planning affect marginalized people, the public, nonhumans, future generations, and historical and culturally significant artifacts. All of these groups deserve consideration, but not equally." (Context urban) (Epting 2017)
Activity:<br><br>Discussion of particular human activities/practices and how they are particularized	Agriculture -> crop breeding, as habitat maintenance, heavy ploughing<br><br>Creation of green corridors<br><br>Human inhabitation<br><br>Low-tech intervention<br><br>Practices generally (unspecified)	"Idealizing a distant wilderness takes our attention away from the landscapes in which we actually live, which are the places where our environmental problems actually begin." (Activity human inhabitation) (Thompson 2012)
Impact:<br><br>Discussion of the type of impact and on whom it falls	Beneficial / harmful / neutral +<br><br>Negative Biodiversity<br><br>Positive Biodiversity<br><br>Cohabitation by opportunists<br><br>Harmful on ecosystem processes<br><br>Enablement of autonomy<br><br>Destructive general (unspecified)<br><br>Landscape restored<br><br>Negative On coral reefs<br><br>Threat to wilderness	"This article challenges Katz's assertion that restoration necessarily implies and involves the domination of nature. It sets out to reveal and critique the assumptions Katz holds in taking such a stance. It considers restoration outcomes in terms of their ability to reinstate value into damaged landscapes" (Impact Landscape restored) (Ladkin 2005)

Approach:<br><br>Theoretical, ontological, or normative key approaches presented	Holism<br><br>Ecocentrism<br><br>Non-Interference<br><br>Non-Dualism<br><br>Third-way environmentalism	"Bailey's environmental vision, on the other hand, reflects a mutualistic orientation, one that links the social and physical well-being and moral virtue of the human community to the beauty and resilience of the cultivated landscape." (Approach Third-way environmentalism) (Minteer 2008)
Proposition:<br><br>Normative proposals or propositions made	No subcodes were generated but proposals were coded each uniquely to retain the message as full as possible.	"Intervention in natural processes, even to 'improve' nature, must be limited, for human action changes natural entities and systems into artefacts. A moral imperative requires respect for the autonomy of nature and resistance to the human domination of nature." (Proposition any human impact on nature changes natural entities into artefacts) (Katz 1993)
Relation:<br><br>Characterizations of human-nonhuman relations, differentiation of relation parties	Care<br><br>Control<br><br>Domination<br><br>Hospitality<br><br>Humility towards nature<br><br>Instrumentalization<br><br>...to land	"Even in such frameworks, however, reciprocal relationships include respecting the personal autonomy of nonhumans. So, whereas influence is not problematic in such paradigms, domination is." (Relations non-domination) (Rohwer 2022)

Thematic results

Wilderness narrative

Wilderness narrative marks **contextually** one of the most central 'birthplace' of contemporary environmental ethics in the context of US preservationism. In wilderness narrative, the attentive orientation is very strongly focused on the landscapes that are considered pristine, 'wild', or 'untouched' by humans. These are often remote and protected areas with strict restrictions to any human presence or activity. Many of those environmental ethics publications that created the 'environmental ethics canon' around the 1980s-1990s involved argumentation, conceptual choices, and other elements that are characteristic of the wilderness narrative. The contextual situatedness of the narrative in the 'wild' was understandable due to the historical roots of the philosophical environmental thought in the US, which had strong linkages to the preservation and the establishment and management of national parks as manifestations of the pristine nature. This was evoked by the worry about the increasing expansion of human activities and their imprint on nature. Thus, many of the discussions representing this narrative also concern forestry and land use, and the expansion of human inhabitation or modernization generally. The more specific topics include the protection and value of wilderness or its re-generation. Amongst the identified narratives, this was the most US-oriented, although the more recent rewilding sub-narrative has gained attention also in Europe.

Scale-wise, the wilderness narrative often includes spatial focus on relatively large wilderness areas: landscapes, ecosystems, habitats, or even biomes. References to specific national parks are common in the more concrete origins of the debate. The temporal scale varies between abstractness, where temporality is absent, to relatively expansive temporal scales in which comparisons are made to 'pre-human', in some (though few) discussions reaching even to previous geological eras (-cenes), or thousands of years: the latter demonstrates deep time in the form of historical ecological baselines, as 'time before human presence', which also strongly impacts the narrative contents. Most attention, nevertheless, is paid to changes within approximately two centuries, especially in the US context.

The wilderness narrative often remains abstracted when discussing human **activities**: it is human life generally, and especially the ongoing societal developments and modernization (or postmodernization, although the word is not used) that are seen as leading to increased land use and the presence of 'human imprint' on nature. When more precise activities are discussed, they often concern the management of protected areas and/or national parks and permitted activities therein (including tourism and game management); restoration; or rewilding in the rewilding sub-narrative, including the "removal of human artefacts and structures, dykes,

levees, wooded banks, canals and other cultural artefacts – that are thought to impede ecological processes" (Drenthen 2018).

In the wilderness narrative, **human presence** is described almost solely as inherently disruptive. This view is quite strongly categorical: humans are either present or absent and their impact then is or isn't there. The presence is not understood as requiring physical presence of humans but climate change, for example, is seen to make every place on earth human-touched. Some differences in degree reveal but mostly in those situations when humans leave different sites 'to be', after which the sites may become more natural again. Viewpoints differ in whether the naturalness and its value can restore after humans leave the place or whether it is permanently lost. This question also manifested itself in the early-stage restoration debate where restoration was criticized as 'faking nature' and that restored environments always include less value than originals: "Thus we might claim that what the environmental engineers are proposing is that we accept a fake or a forgery instead of the real thing. If the claim can be made good then perhaps an adequate response to restoration proposals is to point out that they merely fake nature; that they offer us something less than was taken away." (Elliott 1982). This strong position held that human presence is both unavoidably controlling and makes things artefactual. Thus, it is to be minimised.

The wilderness narrative basically proposes that nature has inherent value in its purity or pristineness; and anything that is of human origin cannot bring all value that nonhuman origin can. The normative implication of this is clear: human presence and any human involvement with nonhuman world should be minimized to the smallest possible. There is no positive, not even neutral, role for human activities: any resource use is seen as land exploitation that necessarily conflicts biocentric values. If humans are present, the best thing they can do is withdraw from places to let nonhumans be. The most extreme statements suggest that 'Intervention in natural processes, even to 'improve' nature, must be limited, for human action changes natural entities and systems into artefacts.' (Katz 1993).

The wilderness narrative involves **strong dualism between nature and culture**. 'Natural' is often given, or implicitly assumed, quite an absolute meaning. This absolute sense of 'natural' would require, for example, Yellowstone to be in a condition with zero mark of human presence or impact; already one human having stepped there or raindrops including any pollutants. However, even such argumentation continues in a way that acknowledges how dependent the evaluation is on the adopted scale: 'Events in Yellowstone can remain 99.44% natural on many a square foot, indeed on hundreds of square miles.' (Rolston 2001).

When the inherently negative impacts of human presence are discussed, the emphasis of **impacts** is highly abstracted: references to degradation, loss of naturalness or intactness, and loss of diversity are common and impacted objects mentioned are either nature generally, landscape, wilderness, or sometimes more specifically a particular type of forest. The differentiation of nonhuman nature into particularized entities is not very common, although there are a few authors who make an exception to this (like Holmes Rolston III) and nature park discussions also involve examples with reference to particular species, especially keystone species in the predator-prey dynamics. When differentiation is made, it retains a holistic approach and the consideration of individual living entities or sentient nonhuman beings with particular concern about their sentience are very rare.

Wilderness narrative differs from others in that the discussion often concerns actually impacts on the **value of nonhuman nature**, not impacts on nonhuman nature or its constituting entities themselves. Some of the argumentation explicitly states that even if ecological restoration managed to restore functions and species compositions identical to the 'pre-degradation' state, impacts regarding values remain degraded and they cannot be restored. This 'value of essences' demonstrates essentialist thinking.

Ethical positions within the wilderness narrative highlight the holistic and inherent value of nature and demonstrate preservationist tendencies. Deontological approaches are common (in both explicit and implicit forms). The moral obligations of non-interference, respect for the autonomy of nature, and a moral obligation to preserve non-artefactual natural value are typical. The narrative involves consequentialist traits too, especially in the rewilding sub-narrative. Amongst the identified narratives, the wilderness narrative makes the strongest distinction between social ethics and environmental ethics and focuses strictly on the latter; exceptions that consider linkages between social and environmental issues are few. For land use ethics, the narrative explicitly demonstrates the 'sparing' position: it is a human obligation to leave as much area as possible aside and set it off for nature, in many cases preferably protecting it.

Rural stewardship narrative

The context of the rural stewardship narrative is centrally agrarian and refers to different types of agricultural contexts from pastoralism to crop production. Forests and forestry are occasionally present. The narrative is situated in rural landscapes where the human presence is frequent and active yet different from urban contexts. The origins of the rural stewardship narrative can be found in the Leopoldian strand of environmental ethical thought that originated around 1940s, 'land ethic', and in many of its treatments within the more contemporary discussions. The narrative represents the rural variant of what is also labelled third-way environmentalism: "In seeking this normative harmonization and practical accommodation of alternative environmental values on the land, I believe Bailey and Leopold fall into a distinct tradition in American environmental thought, a lineage I have elsewhere called "third-way" environmentalism. ... this third way is marked by several features, including an ethically pluralistic approach toward environmental values that defies the dualisms (anthropocentric non-anthropocentric, conservation-preservation, nature-culture, etc.) thrown down by environmental ethicists and historians over the years." (Minteer 2008) Alongside the largely rural focus, the narrative involves frequent engagement with citizenship, civic virtues, and political thought such as green republicanism. The rural narrative has strong US origins, yet its discussion is less explicitly contextualized in the US setting. From the different narratives, the rural stewardship narrative embodies strongest links to the Christian religious tradition, religiously influenced stewardship of the land given 'to Man'[sic], although these features are not represented in the majority of works.

Scale-wise, the rural stewardship narrative is often reflexive of a local or regional spatial scale. This could be characterized as 'a distance that one could go nicely around with a bike'. "Thus we are especially encouraged by some of the work on permaculture by Australians, including Bill Mollison. Working within a bioregion, sharing experiences and knowledge, experimenting with self-reliance and leaving areas of habitat for other species, they are working toward a sustainable and more locally based agriculture. The organic farming techniques publicized by many writers in the context of specific bioregions is another example worth exploring" (Devall & Sessions 1985) Many works in this narrative are sensitive of the regional histories dating back to few decades or a century or two and reflect high awareness of the short-term history about the area and changes in the landscape. Yet, some of the more recent works have also developed the ideas that resonate rural stewardship into spatially and temporally more extended, even on planetary and millennia-long, scales, as 'Earth ethic'.

Human activities appear in this narrative as a concrete and diversely nuanced topic for discussion. They include farming, grazing, small-scale land management such as various soil and landscape management practices; and sometimes forestry. Professional activities are also distinguished and discussed. There is, for example, some remarkably detailed discussion about farming practices and techniques; such details are mostly missing from other narratives. Beyond agricultural and forestry related activities, certain strands of ecological restoration discussions link to this narrative due to their positive view of possibilities for restoration as non-dominating practice that can include aspects of care, respect, and reciprocity.

Human presence is considered as embedded and necessary in landscapes that are by definition characterised by human activities. Humans are viewed as part of the biotic community or ecosystemic land community, its members among other species. The approach to land use leans more to the 'sharing' side of the **'**sharing/sparing**'** paradigms. The role of humans in the biotic community varies from powerful and ir-/responsible members and participants to conservationists, stewards, modifiers and managers: the specification of potentially positive roles is notably rich. Some roles and relations highlight non-hierarchical status while others suggest some level of control and domination. Terms like 'conservationists and stewards' suggest respectful weak control, while modifier-manager roles are such that humans can occupy them 'in good or in bad'. Special attention is given to the agency of farmers.

The rural stewardship narrative **proposes** that ethical cohabitation between humans and other species is possible through care and ecological responsibility that is enacted mostly locally. To discharging responsibilities, professional knowledge and skills are also highlighted. For example, it is seen as important to think in agricultural contexts through soil integrity and how a flourishing soil ecosystem can support food webs.

The narrative suggests that land can be given **holistic ethical regard** that does not exclude production practices for human purposes. The key issue isn't disturbance per se: moderated scales and intervals of disturbance can enhance processes of renewal in soil, as is the case in other ecosystems. "The key issue isn't disturbance per se: moderated scales and intervals of disturbance can enhance processes of renewal in soil, as is the case in other ecosystems." (O'Brien 2020) Even dominion in some forms can be respectful. Reinforcing dualism between management and wilderness is viewed as problematic. Human involvement must not be seen as immediately compromising nature's autonomy. "The exaggeration of human influence over

Earth manifests an anthropocentric narcissism blind to nature's ongoing agency." (Hettinger 2021)

When human **impacts** on nonhuman nature are discussed, they are given significant nuance, which is unsurprising given the 'professionalist ethos' present in this narrative. The discussed types of impacts often focus on biodiversity, land health, soil health, biotic community, and processes. The rural stewardship narrative gives notably more attention to ecological processes compared to other narratives. It is also the narrative where integrity appears as a central guiding normative ideal, due to Leopold's legacy. Health metaphors are common in impact-discussions, especially with regard to soil and land; "...we might consider ourselves healers of a wounded earth" (Hettinger 2021). Attention is also paid to the human impacts on the vitality and species-specific characteristics of species, like behavioural patterns. Nonhuman differentiation is moderate yet varying. Some authors differentiate even species in great detail, producing an ecological outlook to rural environmental ethics. Differentiation of 'classes', rather than individuals, is typical to the rural narrative, and individualistic approaches are rare. Special nodes of differentiation present in this narrative speak of soils and plant ethics.

Rural stewardship narrative reflects place-based, practice-based, and often relational **ethical positions.** Stewardship-thinking is emphasized but there is variation regarding the role and responsibilities of humans. Positions are usually very holist although they also occasionally demonstrate the 'zooming in' into concern for particular individuals at plight and discussing how the responsible management actions benefit individuals of some particular species specifically. One interesting difference to wilderness narrative is the tendency of rural literature to avoid taking stances on the non-/anthropocentrism discussion. This illustrates the 'third-way environmentalism' yet leaves room open to ecocentric positions even if they are not explicitly articulated (due to holism, biocentrism is quite rare). The narrative includes elements from all main ethical-theoretical stances: consequentialism (strong attention to impacts), deontology (principles or duties-based thinking), virtues (of farming or gardening; humility is one example), and relational ethics.

Human interests are not shunned; the argument is that 'good for humans' and 'good for land' actions would often go hand in hand if we truly realized what is good for humans. Consequently, there is often moderate or melding but not strong distinction between social and environmental ethics. The integrative building of connections between human and nonhuman

wellbeing makes the social and environmental concerns integrated every now and then, even if the approach remains nonhuman-oriented.

Knowledge-wise, the rural narrative works give treatment and credibility both to ecology scientific knowledge and to (especially farmers') local knowledge; traditional knowledge, however, is rarely discussed. Knowledge discussions highlight the capacities of a moral agent to observe and read and learn about the environment, suggesting also mutual relations and learning by engaging with nature. "This account is instructive in that it could be seen as indicative of the role nature has in restoring ecosystems. Nature knows how to create a savannah. By paying attention to the patterns of evolving flora and fauna, and through a willingness to re-think pre-formed 'scientific' ideas on the basis of what is really happening, rather than on what should be happening, the inquiring restorer can, perhaps, learn something of these natural creative processes." (Ladkin 2005) The rural narrative marks more interdisciplinary features in the sourcing of scientific knowledge than other narratives, and many of the early authors, such as Aldo Leopold, had multidisciplinary background or interests. This does not permeate the whole narrative though: some works give a stronger role to soil sciences or ecology while other either remain abstract or focus more on farmer knowledge. One interesting feature is the occasional disapproval of modernization in a way that shows critical attitudes towards productivist orientations. Modern knowledge is welcome but not technification in production. In some contexts, modernization is associated with urbanization and urban expansion, which also evokes tensions both within this narrative and in relation to the urban narrative.

Urban cohabitation narrative

Urban cohabitation narrative is **contextually interested** in city spaces but also suburbs, peri-urban areas, city metabolism, and some more general human practices whose visibility and impacts accentuate in urban contexts, such as transport, infrastructure, and building. The urban cohabitation narrative represents another case of non-dualistic 'third-way environmentalism' and marks detachment from dualisms inherent in the wilderness narrative. It is notably different from rural stewardship, however, because works related to urban cohabitation narrative strongly focus on the urban context that gives rise to identifiably different viewpoints and engages in different intellectual traditions, theoretical and conceptual choices, and areas of emphasis in the argumentation. Urban cohabitation narrative also represents a

cross-disciplinary narrative where environmental ethics shows blending with other anthropological, humanities, and social scientific oriented literatures and discussions from political ecology to human geography and justice research in other fields.

Due to the originally strong influence of the wilderness narrative, the urban was for long mainly sidelined from environmental ethics although it had already become a prominent topic in environmental justice discussions oriented on the socio-ecological matters in urban context. While proceeding to these topics was still very slow after the first discussions of bringing urban context into environmental ethics (Gunn 1998) and laments of the 'urban blind spot' (Light 2001) in environmental ethics, the urban context eventually gained attention during 2010s that marked significant change in the level of interest. One of the features that marks the novelty of the urban narrative is the shift of attention away from the classical questions of valuation. "While the push to find or argue for the non-instrumental value of nature can be understood as an important project because it provides a foundation from which environmentalists could argue for the greater protection of natural areas, arguably, focusing on abstract metaphysical and ontological questions, such as intrinsic value, has consequences; consequences such as the ''urban blind-spot'' in environmental ethics." (Noll 2015) Another notable feature is that urban narrative is geographically more diverse than the US-dominated wilderness narrative.

The urban discussion divides into two sub-narratives that are interested in human-nonhuman relations or impacts in urban spaces. One is general environmental ethics and the other one focuses on (sentient) animal ethics and human relations to nonhuman sentients in urban spaces. While there is some overlapping, such as climate change discussions under both perspectives and the concern of individual sentient animals as part of more general urban environmental ethics, the two sub-narratives have many similarities as narratives, yet they rarely enter into dialogue. In this review, we found elements from both sub-narratives.

Spatial **scale** is in urban narratives often city-level. Sometimes focus is on big metropolitan contexts. It is notable that the 'city' size, probably due to the origins of the authors, is often considered so big that many smaller countries would not have any cities, or at maximum one (the capitol), on this comparison. Temporal scale is often missing in urban cohabitation narratives. Many of the approaches under this narrative seem, or at least portray themselves as, relatively ahistorical and atemporal. There are still some exceptions that, for example, compare the historical development of city ideals over time or consider urban development trajectories over the timespan of decades (if rarely for longer). Examples of how future temporality is given attention include discussions regarding climate adaptation and the growing

and status of ‘remarkable trees’ that might start to have hard time ‘fitting in’ the city when they grow.

Urban narrative discusses many human **activities** that vary from dwelling, urban planning and infrastructure design and use (including transport and lighting) to smaller-scale practices such as gardening. Variation between the level of generality or detail is great and urban narrative discussions vary from “modern human life in general” to night lighting in cities. Despite the variation, urban cohabitation narrative is more akin to the rural narrative than to wilderness narrative in the frequency of concrete problems and issues that get discussed. Multispecies entanglements in human activities are commonly present, especially at the smaller-scale level of practices.

Human presence is in the urban cohabitation narratives ‘self-evident’ due to the urban context. The view to human presence is characteristically non-dualist. Humans and nonhumans share built environment. Yet, the narrative remarks difference in the ‘order of sharing’ compared to the rural stewardship narrative: rather than ‘human-in-nature’ (rural stewardship narrative perspective), the perspective tends to be ‘nature-in-city’. While not a chronologically exact depiction, the view often is that in the rural setting ‘nonhumans were there first’ and humans are viewed as newcomers who ought to show respect for the first-order inhabitants of rural landscapes, whereas cities are seen as places that are born only when built. Thus, in a certain kind of genealogical sense, ‘humans were there first’. Exceptions to this perspectival order-shift include discussions that explicitly note how urban land use expansion deprives nonhumans of their habitats.

Human presence is not seen as problematic in itself. Humans are viewed as the modifiers of the environment, which is likened to how other species modify their environments, too. Human presence and modification of city space can be good or bad for other species; there is no presumption about this, except for the occasional admittance that urban constructions do imply land use change that reduces ‘natural environments’. Human presence is seen as active and always evolving; cities are highly dynamic units. The dynamic and constructed nature of urban environments is seen to create a big question of planning that can take better or worse routes from human and nonhuman perspectives. “The work of regional planners has the potential of shifting the design of cityscapes to provide the possibility of integrating green spaces and corridors, while community members and groups actively work to further design and maintain these green areas.” (Noll 2018)

In terms of how human **impacts on nonhuman nature** are discussed, the urban narrative is partly ambiguous yet more detailed especially within the animal-oriented works. Discussions on impacts often combine social and ecological perspectives. Impacts on nonhumans more generally are often discussed in relation to ecosystem health, biodiversity ('diverse nature' or 'endangered nature'), the existence and vitality of nature in general ('ordinary nature'), or the flourishing of some particular nonhuman life forms such as birds, trees, or 'pests' in general. Relational aspects are present in discussions on how, for example, bird life and the planting and growing of (especially native) trees support each other. Oftentimes, the impact discussion shifts perspective from the recipients of moral concern to general environmental qualities: pollution, air quality, and climatic conditions. Attention is paid to more processual impacts on the metabolism of cities, circadian rhythms, resilience, climate change, as well as on structural aspects like the 'animality' of the city and nonhuman mobility. Within the animal-oriented discussions, works differentiate nonhuman inhabitants who serve as particularized examples of more general phenomena. "Loggerhead sea turtles therefore concretely illustrate how ecological light pollution interrupts and negatively affects the functioning and flourishing of biodiverse flora and fauna. As there is a pressing set of reasons to conserve biodiversity, we therefore have at least one, indirect reason to conserve dark nights: natural darkness is, after all, integral to the health and reproductive success of biodiverse ecosystems and their constituents – including the sea turtle." (Dill 2022) Other modes of differentiation include notions how humans categorize and therefore are permitted or required to treat different animal categories differently: duties might depend on liminality, dependence on human resources, and an animal's relationship to human existence in cities, among other factors. Nonhuman climate refugees exemplify a novel differentiation.

The **core propositions** of the urban cohabitation narrative are that nature is not limited to wilderness or reducible to biodiversity: 'ordinary nature', also in urban spaces, matters ethically too. Moreover, human impacts are not categorically good or bad for nonhumans but depend on both degree and toxicity of modification. Integrationist arguments highlight how both human and nonhuman wellbeing would benefit from making cities 'greener'. Urban narrative also suggests that city and nature are not in opposition to each other.

Ethical positions under the urban cohabitation narrative demonstrate cross-disciplinary melding and reach beyond classical environmental ethical distinctions and theories to discuss environmental justice, everyday ethics, and urban political ecology. Partly related to the cross-disciplinary influences, social and environmental are in urban cohabitation narrative often

integrated, and some differentiation of human diversity is often present: at least some social groups, positions, or roles are distinguished among urban inhabitants:

Environmental ethical positions within the urban narrative demonstrate notably often consequentialism and pragmatism. Many of the urban context contributions express or even defend weak anthropocentrism and some works suggest that non-anthropocentrism is an ethically untenable position with regard to urban ethics (Epting 2017). The third-way position and weak anthropocentrism together imply that focus is rarely on whether environments should be managed and modified but *how* this should be done. The urban narrative takes a generally neutral position towards technology as such. Technological solutions, biomimicry, and nature-based solutions are all discussed and viewed as solutions that can be embraced yet require case-by-case evaluation especially in consequentialist terms.

In urban animal ethics, deontological approaches with focus on entitlements and duties, as well as relations of care are more common. Care-relations toward nonhuman entities are important to put into action and we should support urban nonhuman well-being (focus on animals). Hospitality is a good framework for responding to urban wildlife. While most animal ethics approaches to urban context take a stance that implies at least some positive duties to actively assist (non-domesticated) animals in certain situations, most of them suggest the relative distance-keeping for urban co-inhabitants and sort of 'coexistence on parallel planes' in a shared space. Yet, some works make more radical proposals for striving for closer connections and deliberate, more active mutual engagement with urban nonhuman animals.

In terms of **knowledge**, the urban narrative relies much on modern sustainability sciences or general environmental sciences other than biology / ecology; quite little discussion about other types of knowledge. *Is this explained by the context of urban being a 'new setting', i.e. by definition urban areas have not been created or occupied by traditional communities?*. However, discussions about experiences of marginalized groups and their everyday perceptions do exist under this narrative.

Relational narrative

Relational narrative is not a newcomer within environmental ethics: its appearance traces back to the **context of** ecofeminist environmental ethics that emerged in the 1980s. However, the relational narrative resides still in somewhat a periphery. While discussions about

environmental-ethical topics from relational, decolonial, and ecofeminist perspectives are burgeoning vividly, the dialogical relationship to the 'established and acknowledged' tradition of environmental ethics as a distinct discipline is still fragmentary. The decolonial-ecofeminist discussions within this narrative have, anyway, gained significant new momentum with the increasing interest in decolonial perspectives and their incorporation in the academic discussion. Related contributions are contextualised especially in discussions on indigenous territories, global South, and ethics of care (Plumwood 2006; Di Giminiani 2022). (Notably much of ecofeminist literature did not reach our review, however, as it abstains from using concepts that our review relied on and keeps the discussion at a more abstract level of valuations, hierarchies, and societal-structural matters.) Geographically, the relational narrative has authors from diverse origins including Asian and global South countries in much greater representation than in other narratives.

Relational narrative is best understood as a broad umbrella narrative on the basis that relational viewpoints share: criticizing or providing alternatives to the Eurocentric perspectives that have dominated Western philosophical thought; ontological and/or epistemological views that reject the human/nature and reason/emotion dualisms and often embrace ontological pluralism; and weak or strong commitment to relationality, which can be expressed in ontological, epistemic, or ethical terms. However, beyond these shared features and the general 'critical narrative' we acknowledge and identify at least two main sub-narratives. One sub-narrative originates especially in the global South and Latin-American and indigenous contributions and comes with a decolonial and ecofeminist touch. It shows particularly critical attention to global historical relations of oppression that are also seen as permeating academic discourse and engages in strong criticism of mainstream Western philosophical thought. '…the idea of *buen vivir* is not limited to Andean cultural contexts, but is a "useful concept that can support and enhance critical [Western] traditions looking for alternatives to development." Because it is rooted in traditional indigenous culture, Guydnas argues, suma qamaña "questions the very foundations of modernity." It is a multifaceted concept still being constructed.' (May 2017) The other sub-narrative, which originates more in the Asian regions, is non-Western and characteristic of relational and non-dualist ontologies and epistemologies yet puts lesser attention and criticism towards decolonial or structural features.

The spatial **scales** of the relational narrative often focus on regional or territorial landscapes or, for example, areas that are significant habitats for particular indigenous people or traditional local communities. In temporal terms, the strong presence of historical aspects especially in

the decolonial sub-narrative is strong and quite striking compared to other narratives. Ahistorical approaches to ethics are rejected as either unjust or even methodologically or epistemically unsound or inappropriate. Overall, the temporal and spatial situatedness of ethical thinking is highlighted in this narrative.

Relational narrative views **human presence** as constitutive and relational to nature rather than external to it. In some cases, the very conception of nature in the Western understanding is rejected as meaningless. As human relations to their inhabited environments are seen as highly relational and embodied, various activities in concrete forms are given much attention and they vary from farming and collection practices to storytelling and the defence of access rights to land (Di Giminiani 2022). In the decolonial context, the practicing of traditional or indigenous ways of living and cohabitation get much attention. The temporal and geographical aspects of human presence are given more concrete contents instead of treating them as abstractions.

Human impacts can be either good or bad for more-than-human lives. Ethically required or recommended modes of human presence are seen as reciprocal inhabitation of the land community with other fellow members of nonhuman species, or kins; humans can enter into partnerships with kins. The relational narrative also emphasizes the acknowledgement for the agency of nonhuman nature in such discussions. "However, an unfortunate and unnecessary side-effect of the long overdue recognition of the creativity of indigenous humans has been a denial of creativity to nonhuman species and ecosystems—nature scepticism. This latter denial is unhelpful as well as unnecessary because there is no necessary incompatibility between recognizing indigenous (cultural) agency and recognizing nonhuman (natural) agency. A related consequence of the denial of nonhuman agency in the land is the subtle imposition of a land creation story that is not at all culturally neutral but" (Plumwood 2006) Close relatedness to place and nature are characterized as central to this. Especially the decolonial sub-narrative contrasts the ethical mode of human presence against the currently dominant, imperial mode of human presence in the world, shown in mainstream and normalized practices that oppress both humans and other lifeforms. One of the most distinct features of this narrative is the differentiation among various human groups, which relates to the concern paid for social justice, de-/coloniality, and other patterns of domination among humans.

Main propositions under this narrative suggest that land, or landscape, or life in whole (depending on the context and approach), ought to be seen as relational, strongly interconnected, and as kin, or at least as not categorically distinct from human. Attention to relations is more important than object-oriented categorisations, such as labelling species

‘invasive’. Many of the propositions in this narrative distinguish between different types of human relations to land: instead of extractivist relations, relations of care and reciprocity are needed. These calls are also linked to calls for better acknowledging the plurality of knowledges as valid for environmental action but also for the plurality of ethical perspectives. “Rather than starting from abstract first principles (while not discounting the value of such approaches), phenomenological ethics instead focus on how objects come to matter for a perceiver. For Smith, soil’s ethical appearance, provoking certain responses from humans, is a key event. At the same time, there are challenges. Soil’s multiplicity, heterogeneity, and facelessness present several perplexing issues for embodied and phenomenological modes of relational ethics,12 let alone ethical theories based on individualist ontologies.” (O’Brien 2020)

The **ethical positions** emphasize the possibility of positive human presence in landscapes through relational ethics, relations of care, and mutual benefits of interactions to both parties. These standpoints are diverse yet share especially non-dualist and relational views. Environmental and social questions are inseparable. As another variant of third-way, or perhaps fourth-way environmentalism, there is no separable wilderness and environmental and land protection are practices that are also intertwined with daily practices rather than with separate professions. Especially the decolonial-ecofeminist sub-narrative also highlights how the seeking of solutions to ecological challenges must include a broader de-oppression perspective. The decolonial-ecofeminist sub-narrative emphasises affective, ontologically pluriversal, and capitalism-criticizing ethical positions with strong connection to justice discourses.

The relational narrative highlights the diversity of **knowledge** and its sources, giving special emphasis to the place-based and embodied forms of knowing as well as traditional ecological knowledge with relation to, for example, the indigenous groups (Di Giminiani 2022; May 2017. Especially the decolonial-ecofeminist strand of this narrative includes also very critical discussion on the Eurocentric scientific project marked by positivism and rationalism and the misrecognition of other, or plural, knowledges as valuable to seeking solutions to environmental challenges and sustainable cohabitation (Plumwood 2006).

The database of publications included in the review

1. Almassi, B. 2017. Ecological restorations as practices of moral repair. *Ethics and the Environment*, *22*(1), 19-40.

2. Attfield, R. 2014. *Environmental ethics: An overview for the twenty-first century*. Polity Press.
3. Attia, P. 2013. Mega-sized concerns from the nano-sized world: the intersection of nano-and environmental ethics. *Science and Engineering Ethics*, *19*(3), 1007-1016.
4. Barry, J. and K. Smith. 2008. Landscape, politics, labour and identity: Stewardship and the contribution of green political theory. *Landscape Research*, *33*(5), 565-585.
5. Beau, R., 2015. From wilderness to ordinary nature: A French view on an American debate. *Environmental Ethics*, *37*(4), 425-443.
6. Biasetti, P. and B. De Mori. 2020. Towards a conservation ethics. *Iride*, *33*(3), 471-486.
7. Boogaard, B.K., 2019. The relevance of connecting sustainable agricultural development with African philosophy. *South African Journal of Philosophy* 38(3), 273-286.
8. Brister, E., 2019. Unifying Agricultural and Environmental Ethics. *Ethics, Policy & Environment*, *22*(3), 251-258.
9. Cahoone, L., 2009. Hunting as a moral good. *Environmental Values*, *18*(1), pp.67-89.
10. Callicott, J. B. & A. Leopold. 1987. *Companion to A sand county almanac: Interpretive & critical essays*. University of Wisconsin Press.
11. Callicott, J. B. 1999. *Beyond the land ethic: more essays in environmental philosophy*. Suny Press.
12. Cannavò, P.F., 2012. Ecological citizenship, time, and corruption: Aldo Leopold's green republicanism. *Environmental Politics*, *21*(6), pp.864-881.
13. De-Shalit, A. 2003. Philosophy Gone Urban: Reflections on Urban Restoration. *Journal of social philosophy*, 34(1), 6–27.
14. Devall, B. & G. Sessions, 1985. *Deep ecology: living as if nature mattered*. Gibbs Smith Publisher.
15. Di Giminiani, P., 2022. The limits of care: Vitality, enchantment, and emergent environmental ethics among the Mapuche people. *Environmental Humanities*, *14*(2), pp.419-437.
16. Dicks, H., 2017. Environmental ethics and biomimetic ethics: nature as object of ethics and nature as source of ethics. *Journal of Agricultural and Environmental Ethics*, *30*(2), pp.255-274.
17. Dill, K.M., 2022. In defense of wild night. *Ethics, Policy & Environment*, *25*(2), pp.153-177.
18. Drenthen, M., 2018. Rewilding in layered landscapes as a challenge to place identity. *Environmental Values*, *27*(4), pp.405-425.
19. Elliot, R. 1982. Faking Nature. *Inquiry: An Interdisciplinary Journal of Philosophy* 25: 81–93.
20. Enqvist, J.P., S. West, V.A. Masterson, L.J. Haider, U. Svedin, and M. Tengö., 2018. Stewardship as a boundary object for sustainability research: Linking care, knowledge and agency. *Landscape and urban planning*, *179*, 17-37.
21. Epting, S., 2017. On moral prioritization in environmental ethics: weak anthropocentrism for the city. *Environmental Ethics*, *39*(2), pp.131-146.
22. Forbes, W., Antwi-Boasiako, K.B. and Dixon, B., 2014. Some Fundamentals of Conservation in South and West Africa. *Environmental Ethics*, *36*(1).
23. Fox, W. 2006. *A theory of general ethics: Human relationships, nature, and the built environment*. MIT Press.
24. Guha, R. 1989. Radical American environmentalism and wilderness perservation: a third world critique. *Environmental Ethics*, 11(1), 71–83.

25. Gunn, A.S., 1998. Rethinking communities: Environmental ethics in an urbanized world. *Environmental Ethics*, *20*(4), 341-360.
26. Hailwood, S., 2007. Landscape, nature, and neopragmatism. *Environmental Ethics*, *29*(2), pp.131-149.
27. Hailwood, S. 2015. *Alienation and nature in environmental philosophy*. Cambridge University Press.
28. Hettinger, N. 2002. The problem of finding a positive role for humans in the natural world. *Ethics and the Environment*, 7(1), 109-123.
29. Hettinger, N. 2021. Age of Man Environmentalism and Respect for an Independent Nature. *Ethics, Policy & Environment*, 24(1), 75-87.
30. Heyd, T. 2005. *Recognizing the autonomy of nature: Theory and practice.* De Gruyter.
31. Hiernaux, Q., 2021. The ethics of plant flourishing and agricultural ethics: theoretical distinctions and concrete recommendations in light of the environmental crisis. *Philosophies*, *6*(4), p.91.
32. Himes, A., Muraca, B., Anderson, C. B., Athayde, S., Beery, T., Cantú-Fernández, M., ... & Zent, E. 2024. Why nature matters: A systematic review of intrinsic, instrumental, and relational values. BioScience, 74(1), 25-43.
33. Jakobsen, T., 2017. Environmental ethics: Anthropocentrism and non-anthropocentrism revised in the light of critical realism. *Journal of Critical Realism*, *16*(2), pp.184-199.
34. Katz, E. 1993. Artefacts and Functions: A Note on the Value of Nature. *Environmental Values* 2(3), 223–232.
35. Knezović, K., 2009. Environmental-Ethical Aspect of the Evaluation of Plant Gene Technology. *Synthesis Philosophica*, *24*(1), 181-194.
36. Kopnina, H., 2013. Requiem for the weeds: Reflections in Amsterdam city park. *Sustainable Cities and Society*, *9*, 10-14.
37. Ladkin, D., 2005. Does 'Restoration'Necessarily Imply the Domination of Nature?. *Environmental Values*, *14*(2), 203-219.
38. Leopold, A. 1949. *A Sand County almanac, and sketches here and there*. Oxford University Press, USA.
39. May Jr, R.H., 2017. Pachasophy: Landscape Ethics in the Central Andes Mountains of South America. *Environmental Ethics*, *39*(3), 301-319.
40. Mayes, C., 2014. An agrarian imaginary in urban life: Cultivating virtues and vices through a conflicted history. *Journal of Agricultural and Environmental Ethics*, *27*(2), 265-286.
41. McQuillan, A.G., 1993. Cabbages and kings: The ethics and aesthetics of new forestry. *Environmental Values*, *2*(3), 191-221.
42. Michelfelder, D. P. (2018). Urban wildlife ethics. *Environmental Ethics*, *40*(2), 101-117.
43. Millstein, R. 2024. *The Land is Our Community: Aldo Leopold's Environmental Ethic for the New Millennium*. The University of Chicago Press.
44. Minteer, B.A., 2008. Biocentric Farming?: Liberty Hyde Bailey and Environmental Ethics. *Environmental Ethics*, *30*(4), 341-359.
45. Naess, A. 1989. *Ecology, community, and lifestyle: Outline of an ecosophy.* Cambridge University Press.
46. Noll, S. 2018. Nonhuman climate refugees: The role that urban communities should play in ensuring ecological resilience. *Environmental Ethics,* 40(2), 119–134.
47. Norton, B. G. 1991. *Toward Unity Among Environmentalists*. Oxford University Press.
48. O'Brien, A. T. (2020). Ethical acknowledgment of soil ecosystem integrity amid agricultural production in Australia. *Environmental Humanities*, *12*(1), 267-284.

49. Ogden, L.A., 2018. The beaver diaspora: A thought experiment. *Environmental Humanities*, *10*(1), 63-85.
50. Paixão, R.L., 2023. O Direto dos animais às cidades. *Revista de Filosofia Aurora*, *35*, p.e202330416.
51. Palmer, C. 2003. Placing Animals in Urban Environmental Ethics. *Journal of Social Philosophy*, *34*(1), 64-78.
52. Pascual, U., P. Balvanera, C.B. Anderson, R. Chaplin-Kramer, M. Christie, D. González-Jiménez, & E. Zent. 2023. Diverse values of nature for sustainability. *Nature*, 620(7975), 813-823.
53. Paterson, J.L., 2003. Conceptualizing stewardship in agriculture within the Christian tradition. *Environmental Ethics*, *25*(1), 43-58.
54. Peterson, A., 2022. Religion and the Possibility of a Materialist Environmental Ethic. *Environmental Ethics*, *44*(3).
55. Piso, Z., Werkheiser, I., Noll, S. and Leshko, C., 2016. Sustainability of what? Recognising the diverse values that sustainable agriculture works to sustain. *Environmental Values*, *25*(2), 195-214.
56. Raymond, C. M., Anderson, C. B., Athayde, S., Vatn, A., Amin, A. M., Arias-Arévalo, P., ... & Zent, E. 2023. An inclusive typology of values for navigating transformations towards a just and sustainable future. *Current Opinion in Environmental Sustainability*, 64, 101301.
57. Plumwood, V. 1993. *Feminism and the Mastery of Nature.* Routledge.
58. Plumwood, V. 2006a. The Concept of a Cultural Landscape: Nature, Culture and Agency of the Land. *Ethics & the Environment*, 11(2), 115-150.
59. Plumwood, V. 2006b. *Environmental culture: The ecological crisis of reason*. Routledge.
60. Pollock-Ellwand, N., 1994. Cultural landscapes and environmental ethics: The case of puslinch township's historic roadside trees. *Journal of Agricultural and Environmental Ethics*, *7*(2), 189-203.
61. Poole, A. K. 2020. Environmental Philosophy in the City: Confronting the Anti-Urban Bias to Overcome the Human-Nature Divide. In Meagher, S., Noll, S., & Biehl, J. (Eds.): *The Routledge Handbook of Philosophy of the City.* New York: Routledge.
62. Rohwer, Yasha. 2022. "Infringing upon Environmental Autonomy with the Aim of Enabling It." *Environmental Ethics* 44(1): 47–59. https://doi.org/10.5840/enviroethics202251138
63. Rolston, H. 1988. *Environmental ethics: duties to and values in the natural world*. Temple University Press.
64. Rolston III, H. 2001. Natural and unnatural; wild and cultural. *Western North American Naturalist,* 267-276.
65. Rozzi, R. 2016. Global Bioethics and Biocultural Ethics. *Cuadernos de bioética, 27*(91), p. 339.
66. Rozzi, R., F.S. Chapin III, J.B. Callicott, S.T. Pickett, M.E. Power, J.J. Armesto, & R.H. May Jr, (Eds.). 2015. *Earth stewardship: linking ecology and ethics in theory and practice (Vol. 2).* Springer.
67. Saito, Y. 2002. Ecological Design: Promises and Challenges. *Environmental Ethics, 24*(3), 243-261. doi:10.5840/enviroethics200224314
68. Sanford, A.W., 2011. Ethics, narrative, and agriculture: transforming agricultural practice through ecological imagination. *Journal of Agricultural and Environmental Ethics*, *24*(3), pp.283-303.

69. Sarkar, S. and P. Illoldi-Rangel. 2010. Systematic conservation planning: an updated protocol. *Natureza & Conservação*, *8*(1), pp.19-26.
70. Simberloff, D., 2005. Non-native species do threaten the natural environment!. *Journal of Agricultural and Environmental Ethics*, *18*(6), pp.595-607.
71. Simberloff, D. 2009. Moving Beyond Strawmen and Artificial Dichotomies: Adaptive Management When an Endangered Species Uses an Invasive One. *Journal of Agricultural and Environmental Ethics, 22*(1), pp. 73-80. doi:10.1007/s10806-008-9126-5
72. Stone, T. 2018. The Value of Darkness: A Moral Framework for Urban Nighttime Lighting. *Science and engineering ethics, 24*(2), pp. 607-628. doi:10.1007/s11948-017-9924-0
73. Stone, T., 2021. Re-envisioning the nocturnal sublime: On the ethics and aesthetics of nighttime lighting. *Topoi*, *40*(2), pp.481-491.
74. Taylor, P. W. 1986. *Respect for nature*. Princeton University Press.
75. Thompson, A., 2019. Modifying the Myth of the Garden. *Ethics, Policy & Environment*, *22*(3), pp.243-250.
76. Thompson, I. 2012. Landscape and environmental ethics. In Howard, P., Thompson, I., Waterton, E. & Atha, M. (Eds.) *The Routledge Companion to Landscape Studies*, Ch. 38.
77. Thompson, P. B. 1994. *Spirit of the soil: Agriculture and environmental ethics*. Routledge.
78. Thompson, P. 2010. *The agrarian vision: Sustainability and environmental ethics*. University Press of Kentucky.
79. Verharen, C., F. Bugarin, J. Tharakan, E. Wensing, B. Gutema, J. Fortunak, and G. Middendorf. 2021. African environmental ethics: Keys to sustainable development through agroecological villages. *Journal of Agricultural and Environmental Ethics*, *34*(3), 18.
80. Vogel, S. 2015. *Thinking like a mall: Environmental philosophy after the end of nature*. MIT Press.
81. Wang, Z., Fan, M. and Cobb Jr, J., 2020. Chinese Environmental Ethics and Whitehead's Philosophy. *Environmental Ethics*, *42*(1), 73-91.
82. Warren, K. J. 1987. Feminism and ecology: Making connections. *Environmental Ethics*, 9(1), 3–20.
83. Warren, K. 2000. *Ecofeminist philosophy: A western perspective on what it is and why it matters*. Rowman & Littlefield.
84. Westra, L. 1998. *Living in integrity: a global ethic to restore a fragmented earth*. Rowman & Littlefield.
85. Wiebe, J., 2021. Cultural appropriation in bioregionalism and the need for a decolonial ethics of place. *Journal of Religious Ethics*, 49(1), 138-158.
86. Wienhues, A. 2017. Sharing the earth: A biocentric account of ecological justice. *Journal of Agricultural and Environmental Ethics*, 30(3), 367–385.
